# Videolaryngostroboscopy and voice evaluation in patients with rheumatoid arthritis

**DOI:** 10.5935/1808-8694.20120019

**Published:** 2015-11-20

**Authors:** Mario Augusto Ferrari de Castro, Rogério Aparecido Dedivitis, Elio Gilberto Pfuetzenreiter, Ana Paula Brandão Barros, Débora dos Santos Queija

**Affiliations:** a MSc. Lusíada Foundation - Santos (Assisting Surgeon in the Head and Neck Surgery Service of the Santa Casa da Misericórdia de Santos, Ana Costa Hospital. Professor in the Lusíada Foundation Medical School); b Assistant Professor. Supervisor of the Larynx Group in the Department of Head and Neck Surgery of the Hospital of the Medical School of the University of São Paulo (MD); c MSc. Heliópolis Hospital - São Paulo. (Head and Neck Surgeon, Santo Amaro Hospital - Guarujá and Municipality of São Vicente); d PhD - University of São Paulo. (Speech and Hearing Therapist in the Speech and Hearing Therapy of the Ana Costa Hospital in Santos and Heliópolis Hospital in São Paulo); e MSc. - Heliópolis Hospital - São Paulo (Speech and Hearing Therapist - Head and Neck Surgery Service of the Santa Casa da Misericórdia and Ana Costa Hospital in Santos). Hospital Ana Costa e Hospital Guilherme Álvaro, Santos - SP

**Keywords:** arthritis, rheumatoid, laryngoscopy, larynx, voice

## Abstract

Arthritis may affect the larynx and produce symptoms such as hoarseness and vocal fatigue.

**Objective:**

This paper aimed to evaluate the laryngeal manifestations of rheumatoid arthritis.

**Methods:**

This is prospective study assessed 27 patients with rheumatoid arthritis with the aid of videolaryngostroboscopy, auditory-perceptual analysis of the speech using the GIRBAS scale, acoustic analysis and the Voice Handicap Index questionnaire.

**Results:**

Nineteen patients had laryngeal complaints, the main ones being intermittent dysphonia and sensation of a foreign body in the throat. The most frequent laryngoscopical finding was overlapping arytenoids. Three patients had low *pitch*, nine patients had mild dysphonia and roughness. Median acoustic measures were: F0, 198.39 Hz; *Jitter*, 0.815; *Shimmer*, 4.915; and *NHR*, 0.144. Regarding the Voice Handicap Index, the median score was zero in all domains. There was a statistically significant correlation between voice complaints and the domains of this index. Functional classes were significantly correlated to: overlapping arytenoids (*p* = 0.001), *PPQ* (*p* = 0.0257), *Shimmer* (*p* = 0.0295), *APQ* (*p* = 0.0195), and the *VHI* physical (*p =* 0.0227) and total domains (*p* = 0.0425).

**Conclusion:**

Laryngeal complaints were reported by 70.4% of the patients and laryngoscopical alterations were observed in 48% of the subjects. Voice acoustic evaluation and self-perception were altered.

## INTRODUCTION

Since the paper by Mackenzie, in 1894^1^, rheumatism has been correlated to laryngeal involvement and symptoms such as hoarseness and vocal fatigue, and occasionally severe glottic obstruction. In this sense, ankylosis of the cricoarytenoid joint (CJ) was believed to be a much more common disease than what is generally supposed^2^.

Rheumatoid arthritis (RA) may lead to significant distortion and ankylosis of the CJ. The main mechanism that limits joint function is the unilateral or bilateral fixation of the vocal fold by ankylosis consequent to RA. Laryngeal examination may not reveal anything, but it should be done to find out whether there is CJ involvement in RA cases^3^. The following signs have been described: hyperemia and edema of the mucosa covering the CJ; the CJ may become disorganized and the joint ankylosed in a deforming position; and the fixation of one or both vocal folds may occur medially^4^.

These patients may present altered acoustic voice analysis results (disturbed amplitude), a finding connected to the presence of rheumatic nodes on the vocal folds and edema^5^.

Prevalence rates of dysphonia in patients with rheumatoid arthritis ranges between 12% and 27%^6^. The more active rheumatoid arthritis is, the higher is the voice handicap index (VHI) presented by the patients^7^.

In this paper videolaryngostroboscopy was used to assess laryngeal alterations secondary to RA and perceptive-auditory, acoustic, and self-perception of voice handicap tests were applied to analyze voice alterations voice alterations.

## METHOD

This study was approved by the Research Ethics Committee of our institution and given permit n°CEP – 024/2009.

This prospective study enrolled 27 patients with rheumatoid arthritis seen between January and August of 2010. Patients were recruited consecutively in the rheumatology ward of the institution.

The sample consisted of 27 female patients, 23 Caucasians and four non-Caucasians. Ages ranged between 33 and 76 years. One patient was a heavy smoker. Rheumatoid factor blood tests were positive for all patients at some moment in the course of the disease. Table 1 describes patient characteristics.

**Table 1:** Sample distribution based on clinical and demographic variables.

Variable	Category/Measurement	Freq. (%)/Measurement
	Range	33-76
	Median	56
Age year	Mean	55.2
	Standard deviation	10.8
	Range	1-36
RA duration (years)	Median	10
	Mean	12.9
	Standard deviation	9.1
Sjörgen's syndrome	No	22 (81.5)
	Yes	5 (18.5)
	I	8 (29.6)
RA class	II	13 (48.2)
	III	6 (22.2)
Complaint of dysphonia	No	18 (66.7)
	Yes	9 (33.3)

AR: Artrite Reumatóide.

The revised ACR-91 criteria used to categorize the functional status of RA patients are: class I - able to perform usual activities of daily living (self-care, vocational, and avocational); class II - able to perform usual self-care and vocational activities, but limited in avocational activities; class III - able to perform usual self-care activities but limited in vocational and avocational activities; class IV - limited in ability to perform usual self-care, vocational, and avocational activities.

In order to be enrolled in the study, patients had to be at least 20 years old at the time of symptom onset and be categorized on RA functional classes I, II or III (ACR-91). Exclusion criteria included previous orotracheal intubation for 24 hours or more, documented head and neck cancer, larynx surgery, neck trauma, head and neck radiotherapy, central or peripheral neurologic disorders, and having collagenose other than RA.

Patients were required to answer a questionnaire as part of the assessment protocol including the following: 1) Identification data; 2) Data on rheumatoid arthritis (time of disease, association with Sjörgen's syndrome, other associated diseases, functional categorization, extra-articular manifestations, and therapy; 3) Laryngeal complaints. Part of the data was gathered from the patients' charts and from their attending rheumatologists.

Videolaryngostroboscopy was performed using a rigid Karl Storz® de 70° scope connected to a Kay Elemetrics® RLS 9100 B stroboscope and a Toshiba *CCD* (*charge-coupled device)* IK-M41A camera. Images were visualized on a Sony KV-1311 CR video monitor and recorded using a Sony NS67P DVD recorder. Examination was carried out under sustained emission of vowels /e/ and /i/ in modal sounds and at a comfortable volume.

The data sets recorded on DVD were analyzed independently by two ENT and Head and Neck Surgery specialists with experience in laryngostroboscopy. Reports were then analyzed and a mean score was produced and considered as the standard for each studied parameter. The observed parameters were aspect of the free border, glottic closure, predominant glottic cycle phase, vertical level of approximation, range of motion, mucosal wave, phase symmetry, periodicity, arytenoid assessment, hyperfunction, site of mucosal wave, aspect of mucosa, and mucus formation^8^. Additionally, the following vocal fold lesions were considered: telangiectasia^9^, arytenoid edema, bamboo nodes, hyperemia, and reduced vocal fold mobility^10^. Data sets were assessed in a semi-quantitative fashion.

Patients were asked to sustain vowel /a/ in their usual tone and intensity, to count from one to ten, and to produce a segment of connected speech for the purposes of perceptive-auditory and acoustic voice analysis. A Shure® PG 48 professional microphone was used.

Voice acoustic analysis included computer-based acoustic measurements made using software program MDVP (*Multi Dimensional Voice Program)* by *Kay ElemetricS*®. Voice quality was assessed using the GRBASI scale for voice perceptive-auditory analysis^11^^,^^12^. The GRBASI scale is made up of six parameters: *G* - grade of dysphonia; *R* - roughness; *B* - breathiness; *A* - asthenia; *S* - strain e *I* - instability. All parameters were assessed for their absence of presence and degree of severity, namely: 0 - absent; 1 - mild; 2 - moderate e 3 - severe.

The following parameters were considered in acoustic analysis: a) fundamental frequency (F0); b) frequency and amplitude perturbation measurements: jitter (%), PPQ (*pitch -perturbation quotient)*, shimmer (%); and APQ (*amplitude perturbation quotient)*; c) noise measurements: NHR (*noise-to-harmonic-ratio)*; and VTI (*voice turbulence index).*

Pitch was assessed as adequate, low, or high; resonance was categorized as balanced, laryngopharyngeal, or hypernasal; and speech modulation, articulation, and pneumophonoarticulatory coordination were assessed as adequate or abnormal, and the degree of abnormality was graded as mild, moderate, or severe. Parameters were judged by three speech therapists each with over five years of experience in treating patients with dysphonia.

The voice handicap index (VHI) questionnaire was used to assess the subjects' sepf-perception of voice handicap. The questionnaire is divided into three domains - functional, physical, and emotional - each containing ten questions with scores ranging from zero to four, zero being the highest and four the lowest score. Each domain's score is defined by the summation of the answers given by the patients and the final score is defined based on the summation of the scores of the three domains. Final scores may range between zero to 120 points. Thus, the higher the score the more severe is the voice handicap. Scores between zero and 40 indicate mild or absent impact; 41 to 60 means moderate impact; and 61 to 100 suggest severe impact^13^, ^14^, ^15^.

The data gathered from laryngoscopy, selfperceptive and acoustic voice assessment, and VHI were tested for possible correlations with time of RA, RA class, and larynx complaints.

Measures of central tendency and variability were used to describe numeric variables (age, time of RA, acoustic measurements, and VHI) and category frequency distribution (grade of dysphonia, RA class, Sjörgen's, laryngoscopy data, and voice variables).

The Mann-Whitney U test was used to verify the association between numeric variables (measurements) and the groups with two categories, while the Kruskal-Wallis test was used for groups with three categories. Fisher's exact test was used to compare categorical variables on 2×2 tables.

A significance level of 5% was adopted in all statistical tests. Software program STATA version 7.0 was used in statistical analysis.

## RESULTS

Nineteen of the 27 patients had larynx complaints. Symptoms are listed in Table 2. The most frequent finding in laryngoscopy was overriding arytenoids, followed by arytenoid edema (Table 3).

**Table 2:** Subject laryngeal complaints.

Clinical complaint	Freq. (%)
Dysphonia	9 (33.3)
Foreign body sensation	9 (33.3)
Vocal fatigue	7 (25.9)
Xerostomy	7 (25.9)
Dysphagia	3 (11.1)

**Table 3:** Laryngoscopical findings.

Finding	Freq. (%)
Overriding arytenoid	7 (25.9)
Arytenoid edema	4 (14.8)
Anterior cleft	4 (14.8)
Angiodysgenesis	2 (7.4)
Mucus ball	2 (7.4)
Anterior mucus	2 (7.4)
Posterior mucus	2 (7,4)
Rheumatic node	1 (3.7)

Laryngoscopy indicated that only two patients had closure predominantly in the open phase. In terms of overriding arytenoids, five patients had positional asymmetry in adduction. In all cases the glottic wave was situated in the glottis. The mucosal wave was dry in two patients and with edema in four subjects. Overriding arytenoids were the only laryngoscopy parameter statistically correlated to RA class.

In perceptive-auditory assessment, three of the 27 patients had low pitch. Nine had mild dysphonia and roughness. None had asthenia and one had mild breathiness. Six patients had mild vocal strain and five had mild vocal instability. Fourteen patients had balanced and 13 had laryngopharyngeal resonance. Twelve subjects had present phonatory effort (Table 4). Almost all acoustic analysis variables were altered (Table 5).

**Table 4:** Sample distribution based on perceptive-auditory assessment.

Variable	Category	Freq. (%)
	1 (Severe)	3 (11.1)
*pitch*	3 (Adequate)	24 (88.9)
	0 (Absent)	17 (63.0)
G	1 (Mild)	9 (33.3)
	2 (Moderate)	1 (3.7)
	0 (Absent)	17 (63.0)
R	1 (Mild)	9 (33.3)
	2 (Moderate)	1 (3.7)
B	0 (Absent)	27 (100.0)
A	0 (Absent)	27 (100.0)
	0 (Absent)	21 (77.8)
	1 (Mild)	6 (22.2)
_I_	0 (Absent)	22 (81.5)
	1 (Mild)	5 (18.5)
	1 (Balanced)	14 (51.8)
Resonance	2 (Laryngopharyngeal)	13 (48.2)
	1 (Present)	12 (44.4)
Vocal effort	2 (Absent)	15 (55.6)

G: grade of dysphonia; R: roughness; B: breathiness; A: asthenia; S: strain; I: instability.

**Table 5:** Sample distribution based voice acoustic analysis.

Variables (normality)	Measurements	Results
	Range	106.771-266.820
	Median	198.390
FD (150-250)	Mean	200.039
	Standard deviation	32.207
	Range	0.382-3.946
	Median	0.815
Jitter (0.6)	Mean	1.231
	Standard deviation	0.920
	Range	0.224-2.672
	Median	0.435
PPQ (0.4)	Mean	0.691
	Standard deviation	0.566
	Range	1.828-19.333
	Median	4.915
Shimmer (2.0)	Mean	5.696
	Standard deviation	3.445
	Range	1.291-13.103
	Median	3.376
APQ (2.3)	Mean	3.773
	Standard deviation	2.260
	Range	0.098-0.421
	Median	0.144
NHR (0.1)	Mean	0.163
	Standard deviation	0.066
	Range	0.026-0.180
	Median	0.052
VTI (0.0)	Mean	0.057
	Standard deviation	0.030

F0: fundamental frequency; PPQ: *pitch perturbation quotient*; APQ: *amplitude perturbation quotient*; NHR: *noise-to-harmonic-ratio*; VTI: *voice turbulence index.*

Only a few patients reported abnormalities in the complaints related to the vocal handicap index (VHI), but the three domains - functional (VHI-F), physical (VHI-P), and emotional (VHI-E) - were statistically correlated to dysphonia.

Acoustic analysis results were statistically correlated to RA class, PPQ, shimmer, and APQ. RA class was statistically related to the physical domain of VHI and the total VHI score.

## DISCUSSION

Although well known, laryngeal involvement in rheumatoid arthritis is variable and its symptoms less obvious than its pathological manifestations^16^^,^^17^.

In our study, larynx complaints were reported by 19 of the 27 patients (70.4%). The most common symptoms were dysphonia and sensation of a foreign body in the throat, followed by vocal fatigue and xerostomy. There was no statistical correlation between time of RA and the other studied parameters, possibly due to the lack of a linear correlation between functional class and time of disease. Such correlation could have been elicited if the series were larger. Despite the lack of statistical significance, patients with complaints of dysphonia had RA for longer. In acoustic analysis the worse results were seen in patients with RA for longer than 10 years.

Gairola et al.^18^ studied 50 Indian patients with classic RA, 42 females and eight males. Mean duration of RA was 37 years. Most subjects (92%) were on functional classes II and III. Six patients (12%) had symptoms suggestive of laryngeal involvement according to laryngoscopical examination. Mucosal thickening and irritation above the arytenoids were the most commonly seen signs. Only one patient had evidences of acute involvement, with edema and hyperemia in the arytenoids and reduced left vocal fold mobility.

In our series ages ranged between 33 and 76 years (mean age 55.2 years). Only one of the subjects was a chronic smoker. Time of clinical history varied from one to 36 years. Five patients also had Sjörgen's syndrome. In terms of functional categorization (ACR-91^19^), thirteen subjects were on class II, eight on class I, and six on class III. Nine of the 27 patients complained of dysphonia (33.3%).

A wide range of laryngoscopical findings has been described in the literature^20^, ^21^, ^22^, ^23^, ^24^, ^25^. Overriding arytenoids was the main laryngoscopical finding in this study, and five patients had positional asymmetry. Other frequent findings were arytenoid edema, anterior cleft, and angiodysgenesis. No statistical correlation was found between laryngostroboscopy findings and time of disease and complaint of dysphonia. However, a statistical correlation was identified between RA class and the laryngostroboscopy finding of overriding arytenoid cartilages. Indeed, overriding occurs as a consequence of CJ arthritis and ankylosis, and such finding develops as the disease progresses.

Five of the seven patients with overriding arytenoids presented increased phonatory effort, six had increased APQ, and all had increased shimmer, NHR, and VTI.

According to the tests performed, three (11.1%) of the 27 patients had low pitch. Nine had mild dysphonia and mild roughness. None had asthenia or breathiness. Six subjects (22.2%) had mild vocal strain and five had mild vocal instability. Fourteen patients (51.8%) had balanced and 13 (48.2%) had laryngopharyngeal resonance. Twelve subjects (44.4%) had present phonatory effort.

Alterations in vocal acoustic analysis (amplitude disturbances) have been reported in patients with rheumatic nodes and vocal fold edema. In our series, when voice acoustic analysis findings were considered along with RA class (grouping subjects on classes II and III and comparing them to class I individuals),time of disease, and complaint of dysphonia, statistical correlations between functional class and PPQ, shimmer, and APQ were found. Jitter, NHR, and VTI values were higher in patients on functional classes II and III than in subjects on functional class I.

The clinical diagnosis of CJ arthritis is challenging, and is not always connected to disease activity^26^. Other authors disagree, as in their series the more active RA was, the greater was the vocal handicap index presented by their patients^7^. In a certain study, the number of subjects with VHI scores greater than zero and some degree of dysphonia was significantly greater than the number seen in the control group. The exception being the emotional domain, in which the total scores of the control group was greater than that of the case group^6^.

Our study revealed statistical correlations between VHI (physical and total) scores and RA class, and VHI (all domains) scores and complaint of dysphonia. This may be explained by the fact that the VHI is a self-perception vocal handicap index. One of the patients had significantly higher VHI scores when compared to the rest of the series, with possible impacts upon the resulting statistical analysis.

The findings in this study are exploratory. Given the characteristics of the sample, comparisons were made without statistical adjustments and may contain false-positive findings - type I error; the sample size was not calculated and many comparisons may have low statistical power, possibly resulting in type II error.

As an explanation for “omitted diagnosis”, RA patients have many evident generalized signs and symptoms, such as disturbed joints, severe pain related to arthritis in many parts of their bodies, which may shadow the laryngeal symptoms produced by CJ arthritis^3^. Additionally, the sedentary way of life of these patients makes them adapt to the disease's alterations^24^. However, considering state-of-the-art diagnostic tests and the development of a better understanding over laryngeal manifestations of RA, laryngeal involvement by RA is frequent, but not always accompanied by symptoms and apparently not associated with chronic disease.

## CONCLUSION

Complaint of dysphonia was reported by 70.4% of the subjects. Dysphonia and sensation of a foreign body in the throat (33.3%) were the most common complaints. Laryngoscopical alterations were observed in 48% of the patients. Voice acoustic tests and self-perception based on VHI had altered results. Statistically significant correlations were found between complaint of dysphonia and and all VHI domains; disease functional class was correlated with: arytenoid overriding, acoustic measurements of PPQ, shimmer, and APQ, and physical and total domains in VHI.
